# Single-incision laparoscopic appendectomy versus traditional three-hole laparoscopic appendectomy for acute appendicitis in children by senior pediatric surgeons: a multicenter study from China

**DOI:** 10.3389/fped.2023.1224113

**Published:** 2023-07-10

**Authors:** Jie Liu, Guoxian Chen, Xiaowen Mao, Zhihui Jiang, Nannan Jiang, Nan Xia, Aiqin Lin, Guangqi Duan

**Affiliations:** ^1^ Department of Pediatric Surgery, Yijishan Hospital of Wannan Medical College, Wannan Medical College, Wuhu, China; ^2^ Clinical Medicine School of Wannan Medical College, Wannan Medical College, Wuhu, China; ^3^ Department of Medical Biology of Wannan Medical College, Wannan Medical College, Wuhu, China; ^4^ Department of Pediatric Surgery, Maternal and Child Health Hospital of Hubei, Tongji Medical College, Huazhong University of Science and Technology, Wuhan, China; ^5^ Department of General Surgery, Qingdao Women and Children’s Hospital, Qingdao, China; ^6^ Institute of Digital Medicine and Computer-Assisted Surgery of Qingdao University, Qingdao University, Qingdao, China; ^7^ Shandong Provincial Key Laboratory of Digital Medicine and Computer-Assisted Surgery, Qingdao, China

**Keywords:** children, single-incision, conventional, laparoscopy, appendectomy

## Abstract

**Objective:**

The aim of this study was to evaluate the clinical efficacy of single-incision laparoscopy appendectomy (SILA) and traditional three-hole laparoscopy appendectomy (THLA) for the treatment of acute appendicitis in children.

**Methods:**

The clinical data of children (<14 years old) who underwent laparoscopic appendectomy at Yijishan Hospital of Wannan Medical College, Hubei Provincial Maternal Health Hospital and Qingdao Women and Children's Medical Center from January 2019 to June 2022 were retrospectively analyzed. According to the operation method, the patients were assigned to the SILA group or the THLA group. The clinical data, including the efficacy, and the surgical details, including the complications, of the two surgical methods were compared. The personal information of the children and the time of disease onset were recorded.

**Results:**

In this study, the data of 588 patients, including 385 patients in the THLA group and 203 patients in the SILA group were collected. The baseline characteristics between the two groups of patients before surgery were comparable. There was no significant difference in the average operation time between the THLA group and the SILA group (56.31 ± 1.83 min vs. 57.48 ± 1.15 min, *P* > 0.05). There was also no significant difference in the average length of hospital stay between the THLA group and the SILA group (6.91 ± 0.24 days vs. 7.16 ± 0.36 days, *P* > 0.05). However, the FLACC scores of the SILA group (3.71 ± 0.78) were significantly lower than those of the THLA group (3.99 ± 0.56) on the second postoperative day, and the difference was significant (*P* < 0.05). The score of the questionnaire evaluating cosmetic appearance of the postoperative abdomen was significantly higher in the SILA group (15.81 ± 0.36) than in the THLA group (13.10 ± 0.24) (*P* < 0.05). There was no significant difference in the incidence of postoperative complications between the two groups (*P* > 0.05).

**Conclusion:**

SILA is more advantageous in terms of postoperative FLACC scores and cosmetic appearance in children than THLA. There was no significant difference in the incidence of complications or other aspects between the two surgical methods.

## Introduction

Acute appendicitis is highly prevalent in children with an acute abdomen, and appendectomy is still the main method of treatment for this disease (1). With the development of minimally invasive technology, laparoscopic technology has been increasingly used in pediatric surgery (2). The standard treatments for acute appendicitis are early open surgery and laparoscopic surgery. Semm reported laparoscopic appendectomy for the first time in 1983 (3, 4). Compared with traditional open surgery, laparoscopic appendectomy significantly reduces trauma and has been widely used. At present, traditional three-hole laparoscopic appendectomy (THLA) is a common appendectomy technique. However, THLA has a negative impact on quality of life and aesthetics because it requires three surgical incisions. On the contrary, in single-incision transumbilical laparoscopic appendicectomy (SILA), the incision is made around the umbilicus. Because the surgical incision is made around the umbilicus, it is very concealed and could be perceived as a natural scar, thereby achieving good cosmetic results. Although SILA has been increasingly performed in adult and pediatric surgeries, it still has not achieved the expected results (5). Some scholars believe that this technology can not only reduce the appearance of surgical scars but also reduce surgical trauma, relieve postoperative discomfort, and enable patients to move ahead of schedule (6). However, other studies have found that SILA is not superior to THLA in terms of cosmetic outcomes (7, 8).

There is no “golden triangle” operating field in conventional laparoscopy, and the operating instruments create a serious “chopstick” effect in single-site laparoscopy, especially in suturing and knotting operations (9). Therefore, surgeons who are accustomed to conventional laparoscopic operations need to master the “learning curve” of modified laparoscopic techniques (10). The abdominal cavity of children is small, and the operating space is limited, so minimally invasive operations are challenging in such a small space. SILA is infrequently performed in children because it is difficult to coordinate movements with the operation instruments. In fact, whether SILA has obvious advantages over traditional THLA in the diagnosis and treatment of acute appendicitis in children has been controversial. In this study, we retrospectively analyzed the clinical data of children with acute appendicitis in Yijishan Hospital of Wannan Medical College, Hubei Maternal Health Hospital, and Qingdao Women and Children's Medical Center and compared the results with those of traditional THLA. We compared the advantages and disadvantages of THLA and SILA, providing a reference for the clinical treatment of acute appendicitis in children.

## Materials and methods

### Patients and clinical parameters

We retrospectively analyzed the clinical data of children who underwent laparoscopic appendectomy at Yijishan Hospital of Wannan Medical College, Hubei Women's Health Hospital, and Qingdao Women and Children's Medical Center from January 2019 to June 2022 (<14 years). All operations were performed by two senior pediatric surgeons, each of whom had rich experience in performing laparoscopic appendectomy. The surgeon in charge informed the patients about the possible advantages and limitations of the two surgical methods before the operation, and the parents themselves decided the surgical method. We collected the clinical data of the children, including personal information, time of disease onset, surgery details, and complications. Patients with periappendix abscesses, chronic appendicitis or other diseases found during surgery were not included in this study. In this study, acute purulent appendicitis was defined to include both cases of acute purulent appendicitis and acute suppurative appendicitis with perforated appendicitis. Similarly, acute gangrenous appendicitis was defined to include cases of acute gangrene appendicitis and acute gangrene with perforated appendicitis. This study was approved by the Ethics Committee of Yijishan Hospital, Wannan Medical College (No. 2023-LSYD-24), and written consent was obtained from the parents of the children.

### Surgical methods

#### SILA

**Figure 1:**
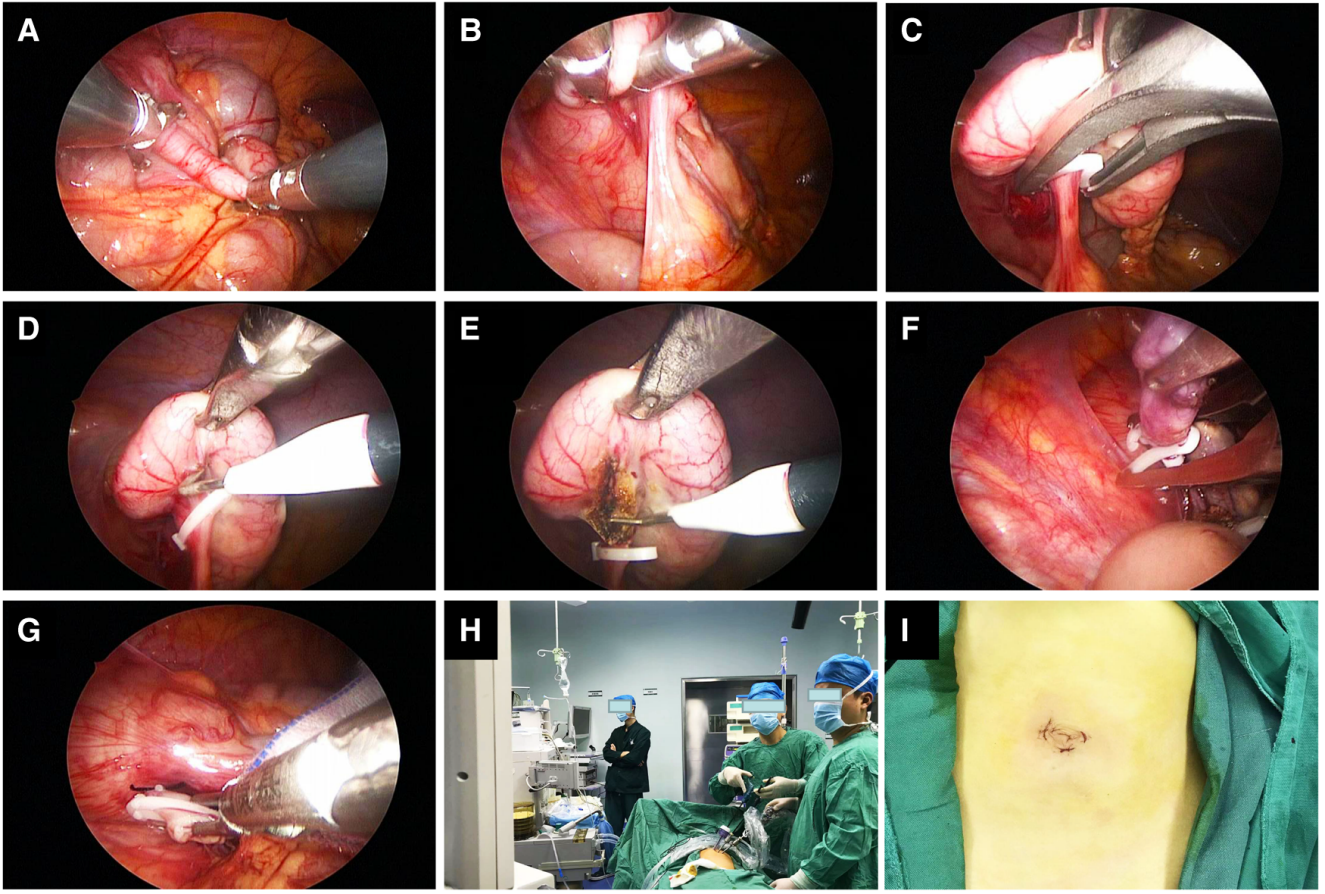
Intraoperative photos and screenshots of single-site laparoscopic appendectomy. (**A–E**) Separate and ligate the mesentery of the appendix. (**F**,**G**) Ligate the base of the appendix. (**H**) The positions of the surgeon and the first assistant surgeon. (**I**) Photos of the postoperative umbilical incision.

The children in the SILA group emptied their bladder before the operation and were given general anesthesia. After the anesthesia was satisfactory, the child was placed in the supine position, the operative field was routinely disinfected, and sterile towels were spread. A transverse incision of approximately 5 mm was made in the lower edge of the umbilicus. A CO_2_ pneumoperitoneum was established with a pneumoperitoneum needle, the intra-abdominal pressure was set to 8–12 mmHg, and a 5 mm trocar was placed through the puncture. The abdominal cavity was routinely explored through trocars placed on the left and right sides of the umbilical ring. The 5 mm and 12 mm trocars were punctured to search for the appendix. The patient was changed from the left-lying position to the Trendelenburg position, with the head down and feet up. The pus in the rectal fossa was drained, the ileocecal part was exposed, the appendix was lifted with grasping forceps, the mesappendix at the root of the appendix was bluntly separated with blood vessel forceps to expose the root of the appendix. Hem-o-lok clips were used to ligate the mesentery of the appendix, and the mesentery was disconnected with an electric hook before undergoing electrocoagulation to stop bleeding. Hem-o-lok clips were used to ligate the root of the appendix twice, and then the appendix was cut off before being removed through a puncture hole in the outer edge of the right umbilical region. The pus that had accumulated in the abdominal pelvic cavity and intestinal space was drained; no active bleeding in the abdominal cavity was observed. Finally, the peritoneum and muscle layers of each puncture hole were sutured with 4-0 absorbable sutures, and the surgical incision was sutured with 5-0 absorbable sutures (Figure 1).

#### THLA

After satisfactory anesthesia in the traditional group, a 5 mm trocar was inserted into the lower umbilical border, and an observation lens was placed. In addition, 5 mm and 12 mm trocars were placed at the anti-Mc Burney's point and the outer edge of the right upper abdominal rectus abdominis, respectively, and the appendix removal procedure was the same as that of the single-site group. In order to minimize postoperative pain, all children were administered local analgesic injections of ropivacaine at a concentration of 0.25% in the surgical incision area.

**Figure 2:**
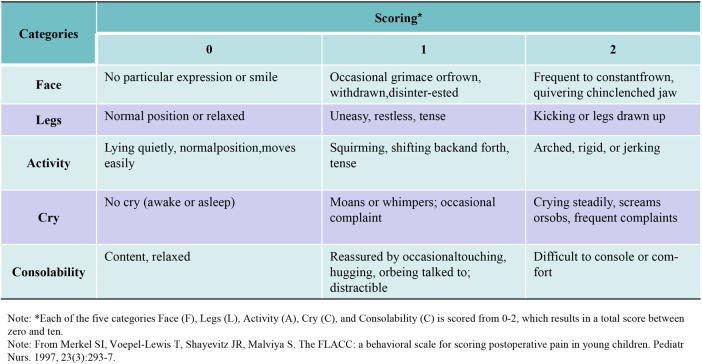
FLACC behavioral pain assessment scale.

The postoperative pain scores were evaluated using the FLACC Behavioral Pain Assessment Scale (11, 12). See Figure 2 for detailed scoring rules.

### Follow-up

Patients were followed up as outpatients or via telephone for 6 months. The parents of the patient completed a questionnaire to assess their perception of the Cosmetic appearance of their child's incision, scores ranged from 0 to 20 (13). The patients were followed up until February 2023.

### Statistical analysis

All statistical analyses in this study were processed using SPSS 23.0 software. The measurement data were expressed as *x* ± *s*, and the Mann‒Whitney test was performed on the count data by *t*-test. A *P* value < 0.05 indicated that the difference was statistically significant.

## Results

### General information

**Figure 3:**
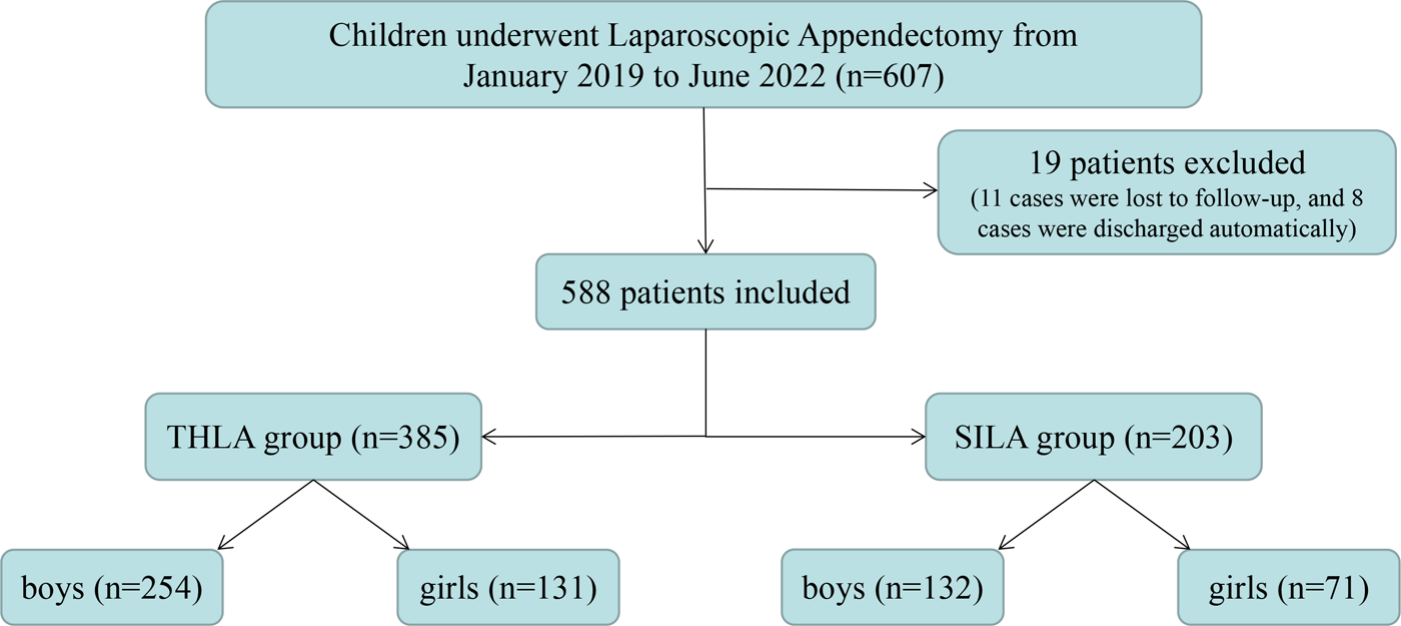
Flow chart of all the patients. THLA, three-hole laparoscopy appendectomy; SILA, single-incision laparoscopy appendectomy.

**Figure 4:**
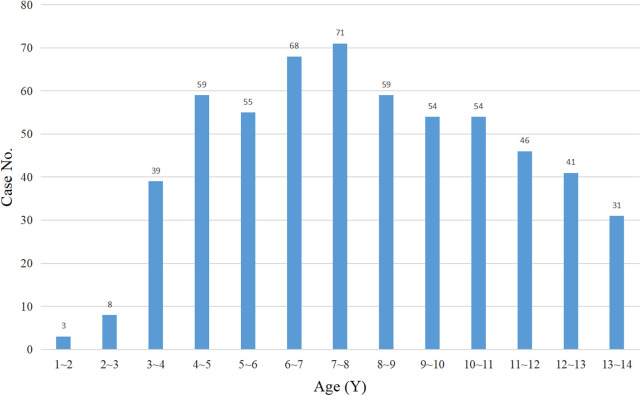
Histogram of patient age distribution in this study.

In this study, the data of 607 patients who underwent laparoscopic appendectomy in three children's surgical centers were collected. Of these 607 children, 11 children did not come to the hospital for re-examination for various reasons and were lost to follow-up. The patients were transferred back to the local hospital for treatment on the second day after the operation. Five hundred eighty-eight patients were finally enrolled in this study, with 385 patients in the THLA group and 203 patients in the SILA group (Figure 3). Laparoscopic appendectomy was successfully performed in all the patients and there was no need for conversion to open surgery. All patients were followed up for 6–24 months after the operation, and the median follow-up time was 10.5 months. The THLA group consisted of 254 boys and 131 girls with a mean age of 7.54 ± 4.23 years. The SILA group included 132 boys and 71 girls, and the mean age was 7.65 ± 2.31 years. The results of the statistical analysis showed that there was no significant difference in sex or age between the two groups (*P* > 0.05) (Table 1). The age distribution of children in this study was analyzed and a histogram was produced (Figure 4). The average duration of abdominal pain in the THLA group was 29.57 ± 8.21 h and that in the SILA group was 28.29 ± 9.38 h. There was no significant difference between the two groups (*P* > 0.05). In the THLA group and the SILA group, the average number of white blood cells in blood tests at admission were 17.03 ± 0.54 × 10^9^ and 16.49 ± 0.47 × 10^9^, the percentages of neutrophils were 82.58% ± 1.23% and 84.13% ± 2.17%, and the CRP levels were 68.39 ± 7.67 mg/dl and 62.62 ± 5.37 mg/dl, respectively. There was no significant difference between the two groups (*P* > 0.05). The postoperative pathology report in the THLA group showed 46 cases of acute simple appendicitis, 210 cases of acute suppurative appendicitis, and 129 cases of acute gangrenous appendicitis. In the SILA group, 13 cases of acute simple appendicitis, 116 cases of acute suppurative appendicitis, and 74 cases of acute gangrenous appendicitis were shown in the postoperative pathology report. There was no significant difference in the pathological characteristics between the two groups (*P* > 0.05). The preoperative clinical characteristics of the two groups are compared in Table 1.

**Table 1:** Demographic data of the THLA and SILA groups before the operation.

Characteristics	THLA	SILA	*t*(*x*^2^) value	*P* value
Age (Years)	7.54 ± 4.23	7.65 ± 2.31	0.423	0.672
Sex				
Boys	254	132	0.053	0.855
Girls	131	71		
Abdominal pain (h)	29.57 ± 8.21	28.29 ± 9.38	0.753	0.452
WBC (×10^9^)	17.03 ± 0.54	16.49 ± 0.47	1.153	0.250
NE%	82.58 ± 1.23	84.13 ± 2.17	1.729	0.084
CRP	68.39 ± 7.67	62.62 ± 5.37	1.161	0.246
Pathological type			4.568	0.102
Acute simple appendicitis	46	13		
Acute suppurative appendicitis	210	116		
Acute gangrenous appendicitis	129	74		

THLA, three-hole laparoscopic appendectomy; SILA, single-incision transumbilical laparoscopic appendicectomy; NE%, neutrophil percentage.

### Surgery-related information

**Figure 5:**
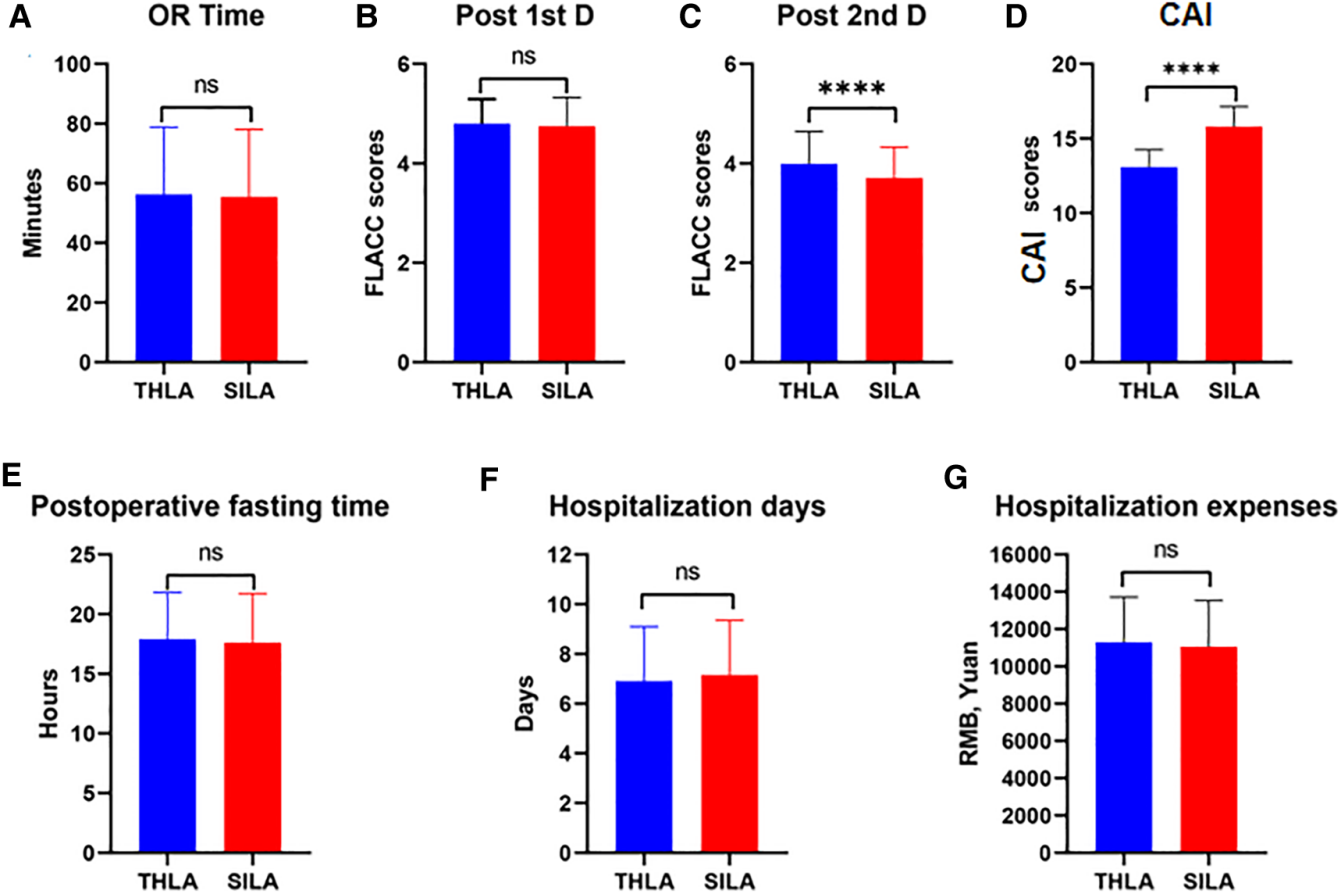
Comparative analysis of three-port laparoscopic appendectomy and single-port laparoscopic appendectomy in this study. ns, not significant; ****P < 0.0001. (**A**) The comparison results between the two groups of Operation time. (**B**, **C**) Comparison of results between two groups of FLACC Behavioral Pain Assessment Scale Scores on the first and second postoperative days. (**D**) Comparative results of Cosmetic appearance of incidence scores between the two groups. (**E**) Comparative results of Postoperative fasting time between the two groups. (**F**) Comparative results of Hospitalization days between the two groups. (**G**) Comparative results of Hospitalization expenses between the two groups.

To study the advantages and limitations of the two surgical procedures, we calculated the operation time of the two surgical methods. The results showed that the average operation time of the THLA group was 56.31 ± 1.83 min, while that of the SILA group was 57.48 ± 1.15 min. There was no significant difference in the operation time between the two groups (*P* > 0.05). The average postoperative fasting time of the THLA group was 17.90 ± 2.36 h and that of the SILA group was 17.61 ± 2.24 h. There was no significant difference in the postoperative fasting time between the two groups (*P* > 0.05). The average hospitalization days of the THLA group was 6.91 ± 0.24 days, and the average hospitalization expenses (RMB) was 11,297 ± 232.6 yuan; however, the average hospitalization days in the SILA group was 7.16 ± 0.36 days, and the average hospitalization expenses (RMB) was 11,065 ± 210.9 yuan. There was no significant difference between the two groups in terms of total hospitalization days and hospitalization expenses (*P* > 0.05). To compare the severity of postoperative incision pain in the children, we collected the FLACC Behavioral Pain Assessment Scale scores from the children's medical records. In this study, only the FLACC scores on the first and second days after surgery were collected because some medical staff did not complete the records on the third day after surgery, and some data were missing. The statistical results showed that there was no significant difference in the FLACC scores on the first postoperative day between the THLA group (4.80 ± 0.16) and the SILA group (4.76 ± 0.42), but the FLACC scores on the second postoperative day were significantly different between the two groups (3.71 ± 0.78). The FLACC scores were lower in the SILA group than in the THLA group (3.99 ± 0.56), and the difference was significant (*P* < 0.05). To further evaluate the patient's satisfaction with their abdominal appearance 6 months after surgery, we administered a survey to parents to assess their perception of the cosmetic appearance of their child's abdomen and found that the cosmetic scores in the SILA group (15.81 ± 0.36) were significantly higher than those in the THLA group (13.10 ± 0.24). The scores were significantly different between the two groups (*P* < 0.05). The results of the analysis are shown in Figure 5 and Table 2.

**Table 2:** Comparison of surgical-related characteristics between the two groups of patients.

Characteristics	THLA	SILA	*t*(*x*^2^) value	*P* value
Operation time (min)	56.31 ± 1.83	57.48 ± 1.15	0.428	0.669
Postoperative fasting time (h)	17.90 ± 2.36	17.61 ± 2.24	0.850	0.396
Hospitalization days	6.91 ± 0.24	7.16 ± 0.36	1.283	0.200
Hospitalization expenses (RMB, Yuan)	11,297 ± 232.6	11,065 ± 210.9	1.103	0.271
FLACC Behavioral Pain Assessment Scale Score				
1st postoperative day	4.80 ± 0.16	4.76 ± 0.42	1.109	0.308
2nd postoperative day	3.99 ± 0.56	3.71 ± 0.78	5.131	<0.0001
CAI scores	13.10 ± 0.24	15.81 ± 0.36	25.58	<0.0001
Postoperative complication				
Wound infection	2/385	2/203	0.427	0.514
Deep space infection	0	0	–	–
Wound seroma	1/385	1/203	0.213	0.645
Postoperative bleeding	0	0	–	–
Urinary retention	2/385	2/203	0.427	0.514
Prolonged postoperative ileus	3/385	2/203	0.067	0.796
Readmission within 30 days	3/385	3/203	0.642	0.423

THLA, three-hole laparoscopic appendectomy; SILA, single-incision transumbilical laparoscopic appendicectomy; CAI scores, Cosmetic appearance of incision scores.

To compare the two groups with the same pathological type of appendicitis, we analyzed the operation time. The findings revealed that the average operation time for acute simple appendicitis was 45.00 ± 18.07 min and 52.68 ± 18.88 min in the THLA and SILA groups, respectively. However, there was no significant statistical difference between the two groups (*P* > 0.05). Similarly, for acute suppurative appendicitis, the mean operation time was 54.50 ± 20.38 min and 55.07 ± 22.18 min in the THLA and SILA groups, respectively. Again, there was no statistically significant difference between the two groups (*P* > 0.05). The mean operation time for acute gangrene appendicitis was 63.49 ± 24.83 min and 64.90 ± 27.07 min in the THLA and SILA groups, respectively, and there was no statistically significant difference between the two groups (*P* > 0.05). The results of the detailed statistical analysis are shown in Table 3.

**Table 3:** Comparative analysis of operation time between two groups based on pathological classification.

Pathological classification	THLA (min)	SILA (min)	*t* value	*P* value
Acute simple appendicitis	45.00 ± 18.07	52.68 ± 18.88	1.687	0.097
Acute suppurative appendicitis	54.50 ± 20.38	55.07 ± 22.18	0.784	0.434
Acute gangrenous appendicitis	63.49 ± 24.83	64.90 ± 27.07	0.905	0.366

THLA, three-hole laparoscopic appendectomy; SILA, single-incision transumbilical laparoscopic appendicectomy.

### Complications

Last, we studied the complications related to the two surgical procedures. Incision infection occurred in 2 patients in the THLA group and in 2 patients in the SILA group, but the difference in the infection incision rates (0.52% vs. 0.99%) was not significant between the two groups (*P* > 0.05). Incision infection was effectively controlled in all children and healed after changing the dressing. Wound seroma occurred in one patient in the THLA group and in 1 patient in the SILA group, and the difference in the wound seroma rate (0.26% vs. 0.49%) was not significant between the two groups (*P* > 0.05). Two children in the THLA group and 2 children in the SILA group developed postoperative urinary retention, and the difference in the postoperative urinary retention rate (0.52% vs. 0.99%) was not significant between the two groups (*P* > 0.05). Children with urinary retention successfully released urine after symptomatic treatment. In the THLA group, 3 children were readmitted due to intestinal obstruction within 30 days after the operation and were cured and discharged after conservative treatment. In the SILA group, 2 children with intestinal obstruction were readmitted within 30 days, and another child with an incision infection was readmitted to the hospital for anti-infection treatment and a dressing change within 30 days. There was no significant difference in the incidence of intestinal obstruction (0.78% vs. 0.99%) or the incidence of readmission within 30 days (0.78% vs. 1.48%) between the two groups (*P* > 0.05). The results are shown in Table 2.

## Discussion

Acute appendicitis is the most common acute abdominal disease in children who are older than 6 years (14). However, in this study, 3 children were 1 year old, indicating that pediatric surgeons should be aware that this disease can present in younger children. The three children's centers involved in this study are renowned for their expertise in treating complex and challenging cases. Typically, local municipal or county-level hospitals prefer to perform appendicitis surgery on older children, while younger children are often referred to the three major medical institutions in this study. As a result, the average age of children in this study was relatively low. Studies have found that the incidence of acute appendicitis in children is gradually increasing. Since the symptoms of appendicitis in children are generally atypical, the disease progresses more rapidly than in adults, and the incidence of perforation is much higher in children than in adults (15). Early surgery is the most important treatment for this disease. Compared with open appendectomy, laparoscopic appendectomy has significant advantages, including a shorter surgical time, a faster postoperative recovery, fewer postoperative complications, and a shorter hospital stay. It may be more suitable for elderly and pediatric patients. With the development of laparoscopic technology, transumbilical single-site, single-port laparoscopic surgery has been increasingly performed in pediatric appendectomy, but whether this technique has more obvious advantages than traditional three-port laparoscopy is still unknown (16). Because single-port laparoscopy requires special operating instruments, which significantly increases medical expenses, its application in primary hospitals in China has been significantly restricted (17). The majority of pediatric surgeons may be more likely to perform SILA instead of THLA. This was a multicenter retrospective study involving three children's medical centers in China and we found that compared with THLA, SILA had better FLACC scores and cosmetic outcomes in children on the second day after surgery. However, there was no significant difference in intraoperative surgery time, hospital stay, total medical costs, or postoperative complications between the two surgical methods.

Laparoscopic appendectomy allows the appendix to be found under direct vision, abdominal exploration and ascites removal, with less trauma, faster postoperative recovery and better cosmetic outcomes (18). It has gradually become the first choice for the treatment of appendicitis. In transumbilical laparoscopic surgery, the navel, a natural biological channel, is the site of entry, thus allowing the postoperative incision to be concealed, which has become the goal of the surgery. Although the abdominal wall is thin, the colon is relatively free, and the umbilicus and root of the appendix are close in children, the incision from the umbilicus is still preferred so that the appendix can be pulled through the umbilical incision with grasping forceps or a homemade copper wire hook. In doing so, appendectomy is completed *in vitro*. However, this technique is only suitable for those with mild appendicitis symptoms, no obvious adhesions to the surrounding structures and a free appendix; this operation significantly increases the risk of an incision infection (19). Using the traditional method, the dissociation of the mesentery of the appendix and the treatment of the root of the appendix are completed under the laparoscope. The appendix is removed through a trocar and placed in a specimen bag. Since the appendix does not touch the wound, the risk of an incision infection is low. Some scholars have suggested that all trocars be placed in the umbilicus and that the appendix should be freed and removed under the laparoscope, which not only ensures a highly concealed incision but also avoids the high risk of an incision infection. The mirror technique is a concern for surgeons (20). Unfortunately, to date, SILA has not been widely recognized for the treatment of acute appendicitis in children (21). Compared with THLA, SILA requires that the spatial relationship between the operating forceps and the observation lens be considered, and the single-incision approach affects the surgeon's and assistant's range of motion, thereby complicating and prolonging the surgery (22). To date, there is no consensus on SILA having more advantages than traditional laparoscopic approaches.

Zhao et al. (23) conducted a meta-analysis of 12 pediatric appendectomy procedures and found that compared with THLA, SILA significantly shortened the hospitalization time, but there was no significant difference in surgical time or other aspects. A meta-analysis conducted by Köhler et al. (24) included a total of 20 studies, and the results showed that extracorporeal SILA seemed to have the advantage of a shorter surgical time, while *in vitro*/*in vivo* SILA showed no significant difference in safety compared to THLA. Aly (25) found that traditional laparoscopic appendectomy is significantly better than SILA in terms of surgical time and intraoperative conversion rate. In addition, some studies suggest that the abdominal wall nearest to the umbilical cord is thin and very close to the cecum. It is possible to slightly expand the umbilical incision and then resect and lift the appendix through the incision under direct vision (26, 27). Lee et al. (28) reported for the first time that in patients undergoing laparoscopic appendectomy, patients with a narrow and deep umbilicus have a significantly increased risk of a postoperative incision infection, while extending the umbilicus incision during surgery seems to reduce the risk of postoperative incision infection. However, in practical practice, pediatric surgeons have found that this method is more commonly used for simple appendicitis, but for complex appendicitis patients with special locations or heavy adhesions, it is very difficult to remove the appendix through an umbilical incision (29).

Some scholars believe that because all operation instruments are used at the same site, the traditional “triangular operation” method cannot be applied smoothly, and there are certain deficiencies in visual field exposure and operational accuracy (30). This viewpoint may greatly limit the development of this technology. In fact, there are certain techniques for placing the three trocars for SILA. The trocar with the lens should be placed as shallow as possible, preferably with the mirror just entering the abdominal cavity, while the trocar with the auxiliary forceps should be placed as deep as possible to minimize the “chopstick effect” and ensure that the surgical operation is completed under direct endoscopy. There are various methods for dissecting the mesentery of the appendix, such as wire ligation, biological clamp closure, and single bipolar electrocoagulation. However, it is difficult to ligate or clamp the root of the mesentery under endoscopy, so an ultrasound knife can be used to directly detach the appendix mesentery. The operation is simple and hemostasis is fast, so there is less intraoperative bleeding and no risk of major bleeding after surgery. In fact, separating the mesentery of the appendix is the most time-consuming process in appendectomy. This study found that the surgical time of SILA was not significantly longer than that of THLA. We consider that this may be related to the fact that most of the laparoscopic appendectomies that were performed at the three pediatric surgical centers included in this study were performed by senior doctors with rich surgical experience who are very familiar with the anatomical structure of the appendix. Therefore, there is no significant difference in the surgical time between traditional laparoscopic surgery and SILA. There are various methods for the treatment of laparoscopic appendiceal stumps, among which the commonly used methods include suture insertion and ligation, biological clamping, and snare thread ligation. These treatment measures are the same for single-site laparoscopic appendectomy and traditional three-hole laparoscopic appendectomy. In addition, this study found that although there was no significant difference in FLACC scores between the two groups on the first day after surgery, the scores of the single site group were significantly lower than those of the traditional group on the second day after surgery, which differed from Que et al.'s (31) study. We believe that the FLACC scoring standard used in this study is more suitable for children. All scores are evaluated and recorded by specialized pediatric surgical staff, and single-site surgical incisions are mainly concentrated in the umbilical region, with no excessive wounds in other areas, which can reduce postoperative pain in children.

This study also has some limitations. First, this was a retrospective study. The specific surgical method was chosen by the family members of the patient before surgery, and all preoperative conversations were initiated by the surgeon. Whether the surgeon's preferred surgery caused any bias remains unknown. Second, although all the surgeries were performed by advanced pediatric surgeons, there may have been some differences in personal skills. Third, The three medical centers involved in this study utilized a single-site laparoscopic appendectomy procedure around the umbilicus due to the high cost of single-port surgical instruments. This is a major consideration for parents and surgeons in developing countries where medical costs can be a limiting factor. The medical staff and parents of the children treated in this study preferred to use the most cost-effective medical equipment to achieve the best surgical outcomes. As China's medical insurance system continues to evolve, the cost of medical devices is expected to decrease, which will enable the use of single-port instruments for more minimally invasive laparoscopic surgeries in future research. Finally, the hospitalization fees are comparable among all three medical centers despite the three medical centers included in this study being in three different provinces in China (Anhui, Hubei, and Shandong). However, there may be slight differences in the charging standards between each province. To avoid these limitations and reduce bias in the future, we will include larger samples and more centers.

## Conclusions

In summary, this study found that SILA has significant advantages over THLA in terms of postoperative FLACC scores and aesthetic outcomes in children. However, there was no significant difference in operation time, length of hospital stay, total medical costs, or incidence of postoperative complications between the two surgical methods.

## Data Availability

The original contributions presented in the study are included in the article, further inquiries can be directed to the corresponding authors.
